# Gut microbiota modulate radiotherapy-associated antitumor immune responses against hepatocellular carcinoma Via STING signaling

**DOI:** 10.1080/19490976.2022.2119055

**Published:** 2022-09-10

**Authors:** Zongjuan Li, Yang Zhang, Weifeng Hong, Biao Wang, Yixing Chen, Ping Yang, Jian Zhou, Jia Fan, Zhaochong Zeng, Shisuo Du

**Affiliations:** a Department of Radiation Oncology, Zhongshan Hospital, Fudan University, Shanghai, China; b Department of Liver Surgery, Zhongshan Hospital, Fudan University, Shanghai, China

**Keywords:** Gut microbiome, hepatocellular carcinoma, radiotherapy, STING, c-di-AMP

## Abstract

Studies of the gut–liver axis have enhanced our understanding of the pathophysiology of various liver diseases and the mechanisms underlying the regulation of the effectiveness of therapies. Radiotherapy (RT) is an important therapeutic option for patients with unresectable hepatocellular carcinoma (HCC). However, the role of the microbiome in regulating the response to RT remains unclear. The present study characterizes the gut microbiome of patients responsive or non-responsive to RT and investigates the molecular mechanisms underlying the differences in patient response. We collected fecal samples for 16S rRNA sequencing from a prospective longitudinal trial of 24 HCC patients receiving RT. We used fecal microbiota transplantation (FMT), flow cytometry, and transcriptome sequencing to explore the effects of dysbiosis on RT. We also examined the role of stimulator of interferon genes (STING) in RT-associated antitumor immune responses mediated by gut microbiota in STING- (Tmem173^−/−^) and cGAS-knockout (Mb21d1^–/–^) mouse models. We propose that primary resistance to RT could be attributed to the disruption of the gut microbiome. Mechanistically, gut microbiome dysbiosis impairs antitumor immune responses by suppressing antigen presentation and inhibiting effector T cell functions through the cGAS–STING–IFN-I pathway. Cyclic-di-AMP – an emerging second messenger of bacteria – may act as a STING agonist and is thus a potential target for the prediction and modulation of responses to RT in HCC patients. Our study highlights the therapeutic potential of modulating the gut microbiome in HCC patients receiving RT and provides a new strategy for the radiosensitization of liver cancer.

## Introduction

The gut microbiome is a complex ecosystem that plays an indispensable role in local and systemic immune responses.^1,2^ As the first line of defense, the innate immune system detects microorganisms or their metabolic products, translates signals into host physiological responses, and activates adaptive immunity.^3,4^ Innate immune responses are based on the recognition of pathogen-associated molecular patterns (PAMPs) through cytosolic DNA sensors, toll-like receptors (TLRs), nucleotide-binding oligomerization NOD-like receptors (NLRs), and other pattern recognition receptors (PRRs).^5^ The disruption of gut bacteria using antibiotics impairs the efficacy of various therapies, including chemotherapy and immunotherapy, suggesting a crucial role of gut-derived PAMPs in the regulation of anticancer immunity.^6,7^ Several preclinical and clinical studies have revealed an interaction between the composition of the gut microbiome and sensitivity to radiation.^8,9^ The role of the gut microbiome in mediating radiosensitivity has generated great interest; however, the mechanisms underlying its influence on radiotherapeutic response remain unclear.

The clinical efficacy of radiotherapy (RT) is attributed to its ability to induce DNA damage, which can result directly in tumor-cell death; however, there is an emerging appreciation for additional antitumor immune responses generated by RT and the remodeling of the tumor microenvironment (TME).^10^ RT-induced DNA damage promotes the activation of the cytosolic DNA sensing pathway mediated by cyclic GMP–AMP (cGAMP) synthase (cGAS) and stimulator of interferon genes (STING). After RT, cytoplasmic double-stranded DNA (dsDNA) stimulates the cGAS-STING pathway, resulting in the production of type I interferons. The efficacy of RT is closely associated with cGAS–STING signaling, as the activation of cGAS and STING is key to generating systemic antitumor immunity. Moreover, innate immune signaling involving cGAS–STING complements the DNA-damaging capacity of RT with the CD8^+^ cytotoxic T cell-mediated destruction of cancer cells.^11^ Specific members of the gut microbiota interact with immune cells to promote tumor clearance, suppress cancer cell metastasis, and inhibit chronic inflammation.^12^ Gut microbiota regulate systemic antiviral immunity via the cGAS–STING–IFN-I axis, promoting host resistance to systemic viral infections.^13^

The liver is the first to be exposed to gut-derived signals, including bacterial products, food antigens, and environmental toxins.^14,^ Gut microbiota communicate bidirectionally via both endocrine and immunological mechanisms. The close relationship between the gut and liver in regulating embryological, anatomical, and physiological processes suggests an important role for the gut–liver axis in the pathogenesis of liver diseases, including nonalcoholic fatty liver disease (NAFLD), which is the third leading cause of hepatocellular carcinoma (HCC) worldwide.^15,16^ Evidence from clinical and preclinical studies suggests that dysbiosis in intestinal microbiota, and consequently, the gut–liver axis may directly and/or indirectly modulate response to RT by remodeling the TME in HCC patients via the cGAS–STING–IFN-I axis.

Understanding how gut microbiota modulate RT-based antitumor immune responses against the HCC liver–gut axis may offer a unique opportunity to inform future efforts in the development of more effective radiosensitization approaches and prognostic markers, Therefore, the present study aimed to describe the relationship between gut microbiota composition and response to RT through the liver–gut axis in patients with HCC and examined the mechanisms underlying gut microbiome disruptions, immune functions, and radiosensitivity. We performed a prospective longitudinal trial in 24 HCC patients receiving RT to investigate the role of the microbiome in regulating patient response to therapy. Using STING- (Tmem173^−/−^) and cGAS-knockout (Mb21d1^–/–^) mouse tumor models, we confirmed that the gut microbiome regulates RT sensitivity in HCC via cGAS–STING signaling in dendritic cells (DCs), which in turn, modulate host cytotoxic T lymphocyte responses. We also demonstrated that cyclic (c)-di-AMP, a bacterial STING agonist, facilitates the RT-induced activation of the cGAS–STING–IFN-I pathway. Our study emphasizes the importance of microbiota in antigen-presenting cell cGAS–STING activation in the TME, which synergizes RT-induced systemic antitumor immune activation. Our results suggest that modification of the gut microbiome through personalized probiotics, prebiotics, or fecal transplantation potentially maximize patient response to RT.

## Results

### Gut microbiome composition is associated with HCC patient response to RT

**Figure 1.**
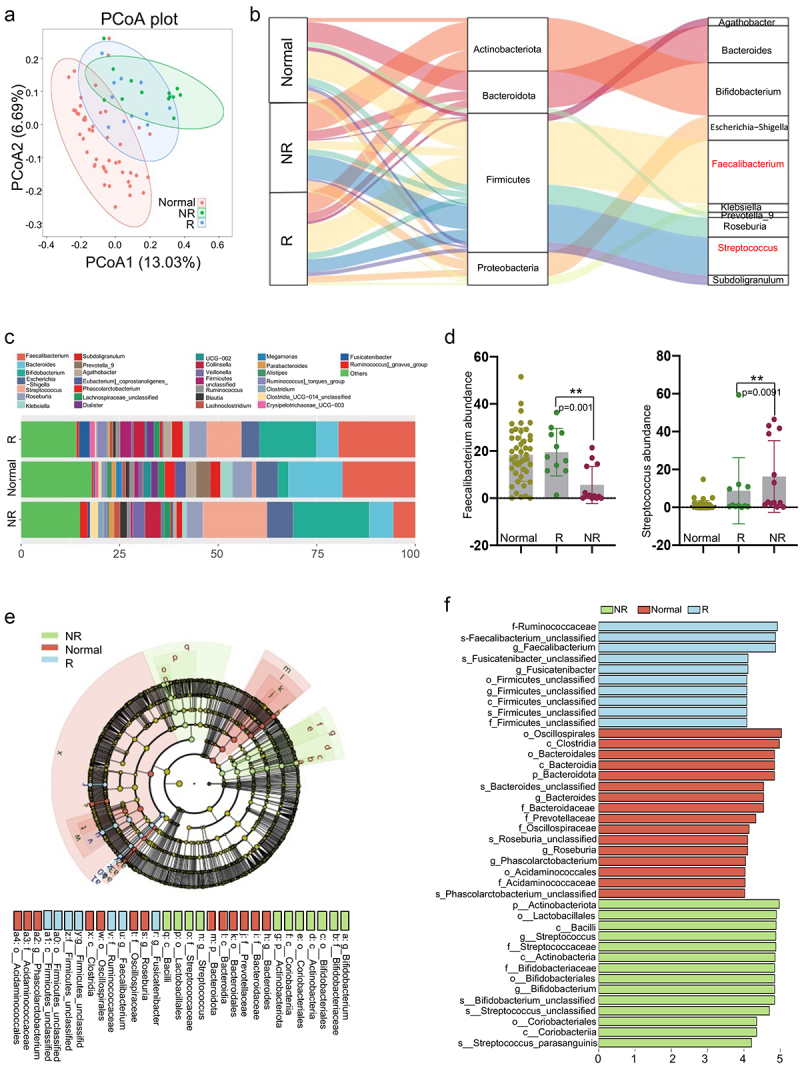
***Gut microbiome composition is significantly correlated with responses to radiotherapy in HCC patients.*** (a) Principal coordinates analysis (PCoA) calculated using Unweighted_unifrac at the OTU-level significantly separated non-responders from healthy individuals and responders; PCoA1 and PCoA2 explained 13.03% and 6.69% of variance, respectively. (b) Average compositions and relative abundances of the bacterial communities in the healthy individuals, R and NR groups at the phylum and genus level. (c) Genus levels of microbial composition in healthy individuals, responders and no responders. Relative abundance of Faecalibacterium (d-left) and Streptococcus (d-right) in healthy individuals, responders and no responders. (e) Histogram of LDA scores for differentially abundant taxa between healthy, R and NR groups, where bar length indicates effect size associated with a taxon. Kruskal-Wallis threshold = 0.05Wilcoxon test threshold = 0.05; LDA score >4. (f) Taxonomic Cladogram from LEfSe, green and red show taxa enriched in the healthy, R and NR groups. Each node represents species classification at this level. The higher the species abundance, the larger the node.

We performed a prospective longitudinal trial in 24 HCC patients who had received RT. The baseline demographic and clinical characteristics of the patients are shown in Table 1. We recruited 46 healthy individuals as controls (6 from the Zhongshan cohort and 40 from public data [PRJNA736821]). The gut microbiome was characterized by 16S rRNA sequencing. The rarefaction curve showed that OTU richness in each sample approached saturation (Figure S1a–c). As estimated by the Chao (Figure S1a and d), Shannon (Figure S1b and e), and Simpson indices (Figure S1c and f), gut microbe diversity was significantly lower in non-responders (NRs) than in healthy controls and responders (according to the best clinical responses determined by RECIST1.1). The observed OTUs in the R group were comparable to those of the healthy controls (Figure S1). A principal coordinate analysis (PcoA) was used to illustrate the microbiome space of different samples, the healthy control, R, and NR groups were distributed in three distinct clusters, representing significant differences in gut microbiome polymorphisms (Figure 1a). Compared to the NR group samples, the distribution of the R group samples was closer to that observed for the healthy control group samples; this indicates greater similarities in the microbial community between these two groups. These findings imply that serious dysbiosis in the gut microbiome of HCC patients is related to responsiveness to RT.

**Table 1.** Clinical characteristics of HCC patients enrolled in this study.

Characteristics	R	NR	*p*-value
Age			
Mean ± SD	56.45 ± 5.17	61.46 ± 14.33	.28
Gender			
Male	10	12	
Female	1	1	1.00
HbsAg			
Positive	10	13	
Negative	1	0	.46
Tumor size			
Mean ± SD	6.23 ± 3.26	5.74 ± 2.81	.70
Number of Tumors			
Single	7	5	
Multiple	4	8	.42
BED (Gy)			
Mean ± SD	65.00 ± 6.46	64.53 ± 7.28	.87

Patient characteristics, BED:Biological Equivalent Dose

To identify the microbial markers associated with RT efficacy, we compared the taxonomic composition of the gut microbiome between the R and NR groups. Firmicutes, Actinobacteria, Bacteroidetes, and Proteobacteria were the dominant phyla in all samples (Figures 1b and S2a). At the genus level, *Faecalibacterium* was significantly enriched in the healthy control and R groups (*p* = .0010), Figure 1 (b–1) consistent with previous reports regarding CTLA-4 and PD-L1 blockade.^6,17^ Meanwhile, members of the genus *Streptococcus*, which are involved in the development of metabolic disorders, diabetes, and colon cancer,^18^ was more abundant in the NR group (*p* = .0091), Figure 1 (b–1). The bacterial compositions at the phylum and family levels are shown in Figures S2a and S2b, respectively. We used a linear discriminant analysis effect size (LEfSe) to assess the difference in community structure between the R and NR group and identify the species associated with radiosensitivity. The members of the order Clostridiales, family *Ruminococcaceae*, and genus *Faecalibacterium* showed greater abundance in the R group; members of the order Lactobacillales were more abundant in the NR group (Figure 1e,f). These results provide empirical evidence that different gut microbes may have distinct effects on the efficacy of RT and prognosis of HCC patients; however, this finding must be validated in larger cohorts.

### The gut microbiome regulates radiosensitivity via the modulation of host cytotoxic T-lymphocyte levels

**Figure 2.**
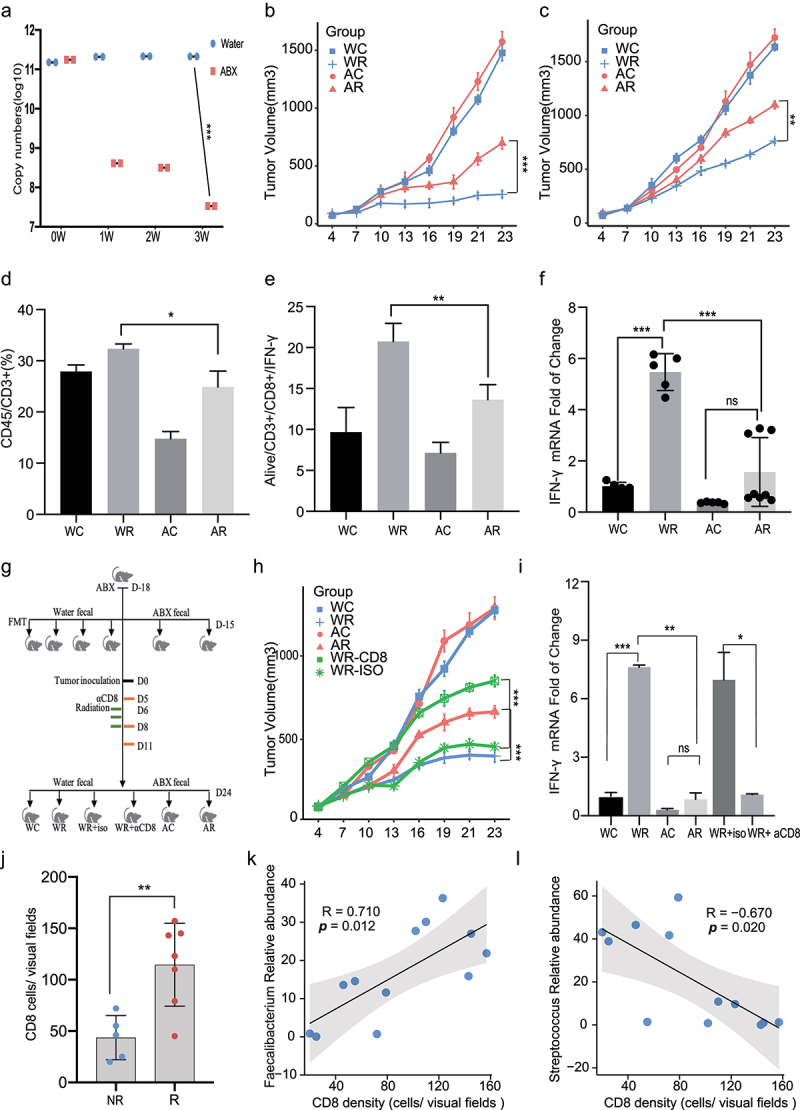
***Dysbiosis of gut microbiome impairs the efficacy of radiotherapy by downregulation of host antitumor CTL cell responses.*** (a) Copy numbers of bacterial 16S rRNA gene in feces were determined by real-time PCR before tumor implantation. (b) Representative H22-OVA tumor growth curves of Water or ABX treated mice during radiotherapy. (c) Abscopal effects of H22-OVA tumor growth curves of Water or ABX treated mice during radiotherapy. Analysis of tumor-infiltrating CD3+ T cell population (Live/CD45+/CD3+cells) (d) and CD8/IFN-γ + population (Live/CD45/CD3/CD8/IFN-γ cells) (e) using flow cytometry. (f) Quantification of IFN-γ mRNA by qRT-PCR in tumors. (g) Experimental designs of FMT and CD8 delete studies: FMT was performed after 3 days of ABX treatment. Two weeks later, H22-OVA cells were inoculated. For the CD8 depletion assay, anti-mouse monoclonal CD8-blocking antibody or isotype control mAb were intraperitoneally injected once every 3 days from the first day before radiotherapy (Day 5). (h) Representative tumor growth curves of groups as mentioned above. (i) Quantification of IFN-γ mRNA levels in tumor tissues by qRT-PCR. (j) Quantification by IHC of tumor infiltrating CD8+ T cells in R (n=7) and NR (n = 5) groups (p=0.0094). Correlation analysis between tumor infiltrating CD8+ T cells and Faecalibacterium (k) as well as Streptococcus (l) abundance in the gut. Representative data shown in A to I were conducted with 4 to 7 mice per group. Data are represented as mean ± SEM. *P < 0.05, **P < 0.01, ***P < 0.001and ns No significant difference.

To elucidate the relationship between HCC radiosensitivity and the gut microbiome, we used an antibiotic cocktail to establish gut-microbiome-elimination mouse models (“ABX” tumor group) and used water as the control solution (“Water” tumor group). After 3 weeks of antibiotic treatment, the mice in the ABX group exhibited an abnormal expansion of the cecum (Figure S3a) and a rapid decline in copy numbers (Figure 2a) of gut bacteria, with no significant effects on body weight (Figure S3b). The mice were subcutaneously injected with murine H22 tumor cells and received clinically relevant fractional radiation doses when the tumors reached >80 mm^3^ in size. ABX treatment significantly counteracted the antitumor effects of RT on target and abscopal tumors (Figure 2b,c) by suppressing radiation-induced apoptosis and proliferation, which was accompanied by the downregulation of cleaved caspase 3, cleaved PARP, and BAX (Figure S4a–c). In an intrahepatic orthotopic tumor model, we also found that gut dysbacteriosis markedly counteracted on the efficacy of RT (Figure S4d).

We evaluated the relationship between the gut microbiota and the immune microenvironment during RT by flow cytometry and immunohistochemistry and found that gut microbiome dysbiosis significantly impaired RT-induced T cell infiltration (Figures 2d and S5a, b). Meanwhile, the infiltration of active CD8^+^/IFN-γ^+^ T cells in the tumors from mice in the ABX and ABX+IR groups was significantly suppressed, compared to that in the tumors from mice in the Water and Water+IR groups, respectively (Figures 2e and S5c). ABX treatment suppressed the IFN-γ mRNA (Figure 2f) and protein (Figure S5d) levels. To confirm the essential roles of gut microbiota in determining the efficacy of RT, we performed fecal microbiota transplantation (FMT) before tumor implantation (Figure 2g). The prophylactic transfer of fecal water, but not ABX fecal water, into ABX mice was sufficient to restore the tumor elimination effects of RT (Figure 2h) and rescue the IFN-γ levels in the tumor tissues (Figure 2i). The rescue effects of FMT were further abrogated in CD8-depleted mice (Figure 2h,i). The roles of the gut microbiome on the efficacy of RT in HCC are therefore dependent on intratumoral CD8^+^ T cell accumulation.

We evaluated the degree of tumor-associated immune infiltration in HCC patients and observed a higher density of CD8^+^ T cells in the R group (n = 5) than in the NR group (n = 7) (*p* = .0052), Figures 2j and S5e. Using pairwise comparisons, we found that the intratumoral infiltration of CD8^+^ T cells was positively correlated with the abundance of members from the genus *Faecalibacterium* (R = 0.710, *p* = .0012) and negatively correlated with the abundance of members from the genus *Streptococcus* (R = – 0.670, *p* = .020; Figure 2k,l). These results indicate a potential mechanism by which the enrichment of specific bacterial taxa modulates the response of tumor-specific T cells after RT for HCC.

### Gut dysbacteriosis inhibits the activation of IFN-I-related pathways and downregulates antitumor immunity

**Figure 3.**
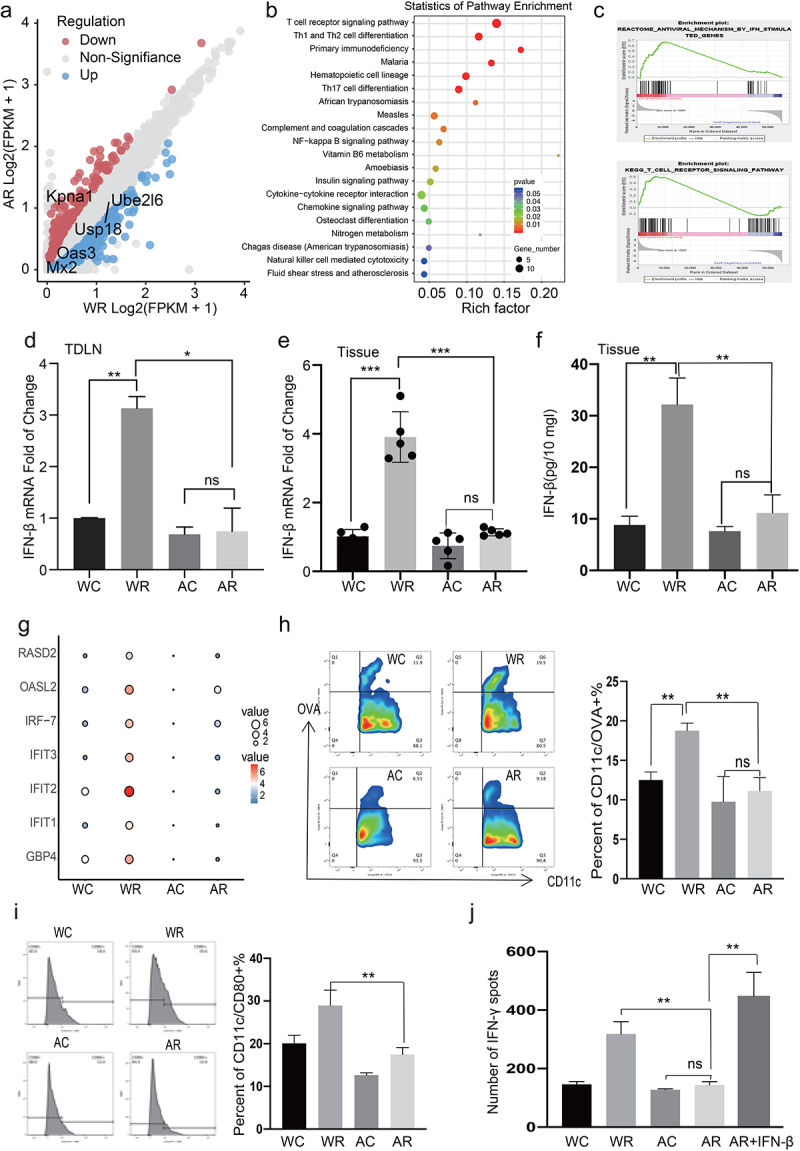
***Dysbacteriosis inhibits interferon I*** ***related pathways and down-regulates antitumor immunity***. (a) A total of 469 differentially expressed genes were identified by RNA-seq analysis in Water and ABX irradiated tumors. (b) KEGG pathway analysis of differentially expressed genes (FC>2 or FC<0.5 and p value < 0.05) in Water and ABX irradiated tumors. (c) GSEA analysis of Apoptosis and IFN-related pathway. Quantification of IFN-β mRNA by qRT-PCR in tumor draining lymph nodes (d) and tumors (e). (f) Concentrations IFN-β in tumors were measured by ELISA, and values expressed as pg/10 mg of tumors. (g) Quantification of interferon stimulated gene (ISG) mRNA by qRT-PCR in tumor draining lymph nodes. (h-i) Analysis of antigen specific dendritic cells (DC) (CD45+/CD11c +/ SIINFEKL+ cells/CD80) in TDLN by flow cytometry. (j) Purified CD11c+ DC cells were co-cultured with naive CD8+T cells with or without IFN-β (10 ng/ml), and IFN-γ secretion was detected by ELISPOT assay. Representative data shown in D to J were conducted with 5 mice per group. Data are represented as mean ± SEM. *P < 0.05, **P < 0.01, ***P < 0.001and ns No significant difference.

To explore the mechanisms underlying the differences in the response to RT, we performed an RNA-seq analysis of irradiated tumor tissues harvested from the mice in the Water and ABX groups. A pathway enrichment analysis of 469 differentially expressed genes revealed that T cell receptors and differentiation, nuclear factor-kappa β signaling, natural killer cell-mediated cytotoxicity, and cytokine−cytokine receptor interactions were significantly enriched (Figure 3a,b). A gene set enrichment analysis (GSEA) revealed that the IFN-stimulated, apoptosis, chemokine signaling, and T cell receptor signaling pathways were positively co-enriched in the healthy gut microbiome, whereas the anti-inflammatory peroxisome proliferator-activated receptor-γ (PPAR) signaling pathway was negatively co-enriched (Figures 3c and S6a). Moreover, gut dysbacteriosis was associated with markedly reduced IFN-β and -α levels in both tumor-draining lymph nodes (TDLNs; Figures 3d and S6b) and tumors (Figure 3e,f). The type I IFN-mediated induction of the expression of interferon-stimulated genes (ISGs) also decreased significantly in the ABX-treated mice (Figure 3g).

The antitumor efficacy of RT reportedly relies on the IFN-stimulated pathway and cross-priming of tumor-associated DCs.^11^ Therefore, we evaluated the levels of antigen-specific DCs (CD45^+^/CD11c^+^/SIINFEKL^+^ cells) in TDLNs by flow cytometry. Gut microbiome dysbiosis significantly suppressed the proportion and maturation rates of DCs, consistent with the findings of previous studies (Figure 3h,i).^19^ To determine whether functional differences in DCs could explain the differences in T cell priming observed *in vivo*, we purified a CD11c^+^ population from TDLNs and conducted a cross-priming assay *in vitro*. Our results verified that ABX treatment suppressed the local antigen-presenting ability of CD11c^+^ DCs and led to decreased IFN-γ levels (Figures 3j and S6c). To establish whether the attenuated cross-priming ability of DCs was due to impaired IFN-I secretion, we performed a rescue assay in which we co-cultured CD11c^+^ DCs with naïve CD8^+^ T cells in the presence of IFN-β and found that the impaired antigen presentation abilities of the DCs were restored (Figures 3j and S6c). Therefore, the dysbacteriosis-mediated suppression of IFN-I suppressed the activation of adaptive immunity, which impaired the antitumor efficacy of RT against HCC.

### The gut microbiome regulates RT sensitivity in HCC via cGAS–STING signaling in DCs

**Figure 4.**
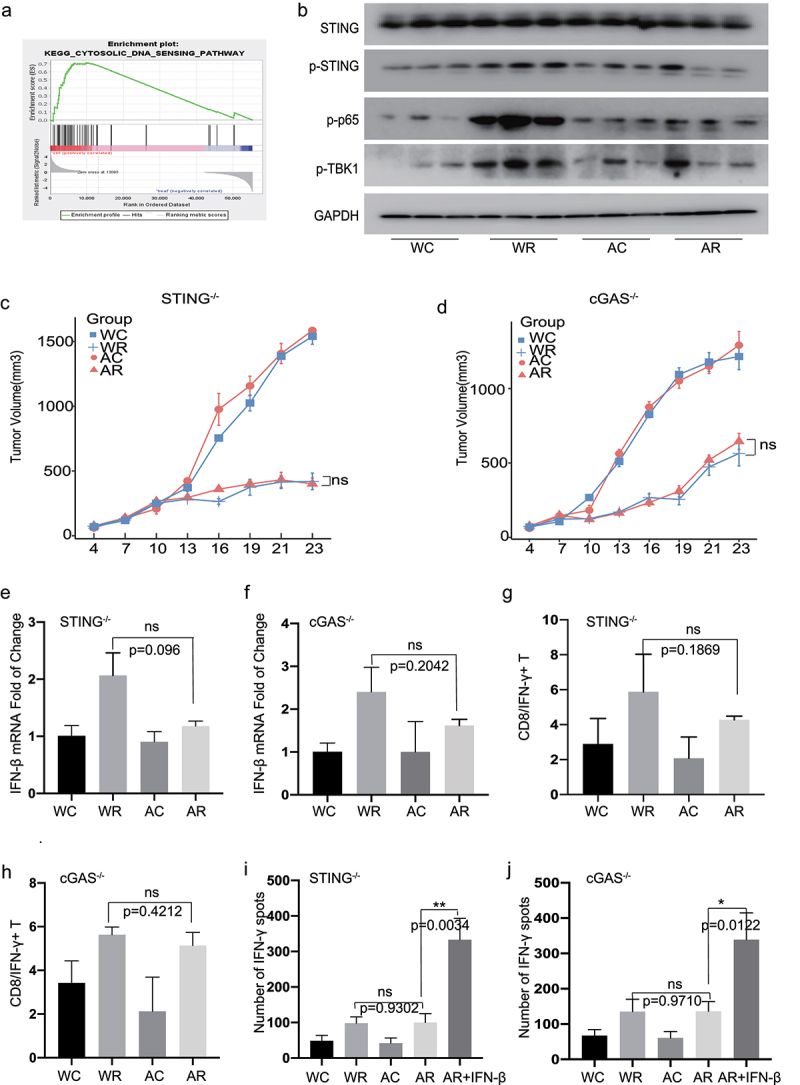
***Immune regulation of gut microbiome are depended on the cGAS /STING signaling pathway in the HCC radiotherapy.*** GSEA analysis of Cytosolic DNA sensing pathway (p = .087, NES = 1.55). (b) Western blot analysis of STING, p-STING, p-p65, p-TBK1. Representative H22-OVA tumor growth curves of cGAS-/-(d) and STING-/- (c) mice during radiotherapy. Quantification of IFN-β mRNA by qRT-PCR in tumor draining lymph nodes of cGAS-/-(f) and STING-/- (e). Analysis of tumor-infiltrating CD8/IFN-γ + population (Live/CD45/CD3/CD8/IFN-γ cells) in cGAS-/-(h) and STING-/- (g) mice by flow cytometry. IFN-γ ELISPOT assay was performed in cGAS-/- (j) and STING-/- (i) mice. Representative data shown in B were conducted with 3 mice per group. Representative data shown in C to J were conducted with 5 mice per group. Data are represented as mean ± SEM. *P < .05, **P < .01, ***P < .001 and ns No significant difference.

To elucidate the mechanistic interplay between the gut microbiome and DCs, we purified CD11c^+^ DCs from mice in the Water and ABX groups for the genome-wide transcriptional sequencing of single-species cells. The GSEA revealed a marked suppression of DNA-sensing signaling in ABX-treated mice (Figure 4a). The cGAS-STING pathway, a major sensor of cytosolic DNA, induces the production of type I IFNs and other inflammatory cytokines upon the recognition of self or endogenous pathogenic DNA.^20^ Consistent with the RNA-seq data, a western blot analysis confirmed that the cGAS–STING pathway was suppressed in ABX-treated mice, as shown by the decreased phosphorylation levels of STING, p65, IRF3, and TBK1 (Figure 4b).

The cGAS–STING pathway regulates IFN-I expression via RT-mediated antitumor effects. However, the direct relationship between the cGAS–STING pathway and host immune regulation mediated by the gut microbiome after RT has not yet been determined. We compared radiosensitivity and antitumor immune responses in Tmem173^−/−^ (STING-knockout) mice from the Water and ABX groups. Compared to wild-type mice, the diminished efficacy of RT due to antibiotic administration was abolished in Tmem173^gt^ mice (Figure 4c). Moreover, the dysbacteriosis-associated suppression of IFN-β production (Figure 4e) and CD8/IFN-γ^+^ T cell infiltration (Figure 4g) observed after RT was reversed. Next, we purified CD11c^+^ cells from STING^–/^^–^ TDLNs and performed an IFN-γ ELISPOT assay, as described in wild-type mice. The STING deletion abrogated the ABX-mediated impairment of DC cross-priming. Furthermore, exogenous IFN-β supplementation restored the ability of STING-deficient DCs to cross-prime specific T cells (Figure 4i and S7a). Similar patterns were observed in cGAS-deficient Mb21d1^–/^^–^ mice (Figures 4d,f,h,j and S7b).

To confirm the role of cGAS–STING signaling in the gut microbiome-mediated immune regulation of clinical responses, we administered the stools of RT-treated R and NR patients to ABX-treated mice by gavage (Figure S7c). FMT from the R but not NR patients caused a significant delay in tumor growth after RT in the wild-type mice, but this tumor growth-suppressive effect disappeared in cGAS- and STING-knockout mice (Figure S7d). These results suggest that RT-induced cGAS–STING signaling in DCs is regulated, presumably, by the metabolites produced in the gut microbiome.

### C-di-Amp, a bacterium-derived mediator, is involved in the regulation of sensitivity to RT in HCC

**Figure 5.**
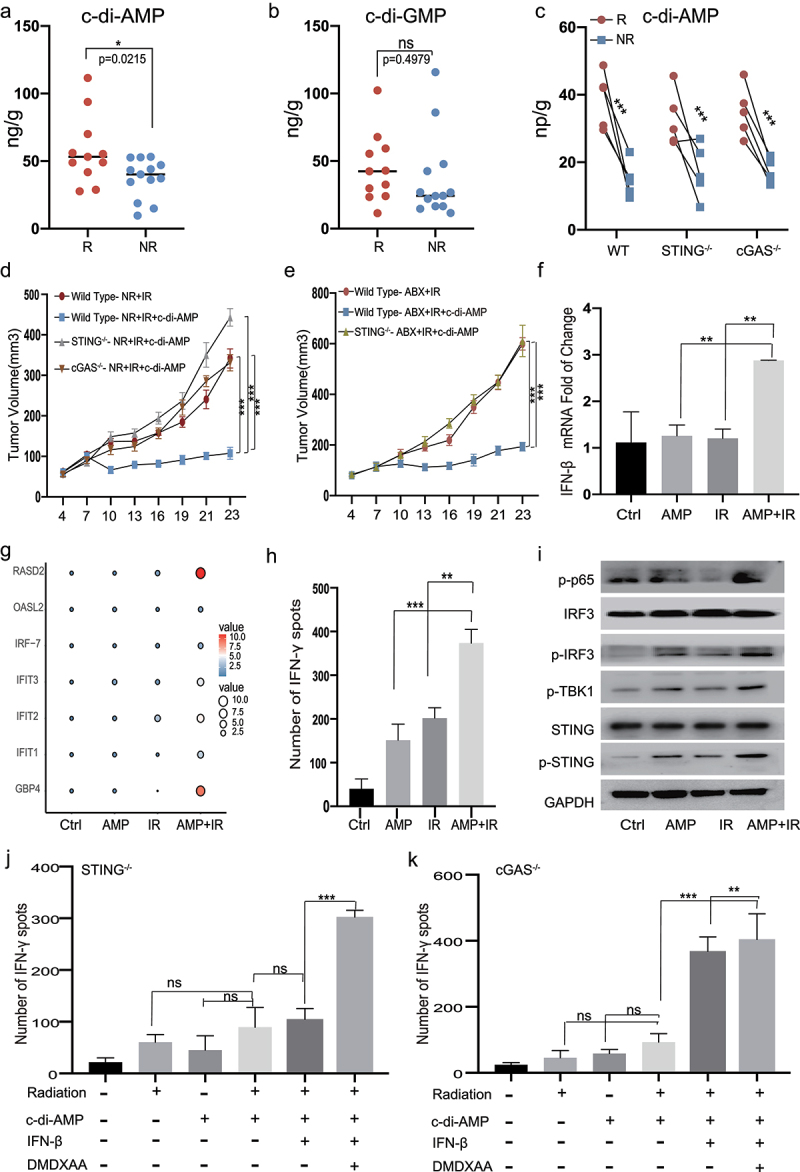
***Combination of c-di-AMP with radiation promotes the secretion of IFN-β and IFN-γ.*** UPLC/MS analysis of c-di-AMP (a) and c-di-GMP (b) levels in fecal samples from HCC patients (n = 24). H22 cells were exposed to 8 Gy radiation, and culture supernatants collected after 24 h. (c) After FMT with R and NR patients, the content of c-di-AMP was detected by UPLC/MS. (d, e) Representative H22-OVA tumor growth curves of cGAS-/-, STING-/- and wild type mice during radiotherapy after FMT and intraperitoneal (i.p.) administration of c-di-AMP. (f, g) BMDCs were stimulated with irradiated tumor-supernatants, or c-di-AMP (5 ug/ml) or a combination for 48 h on day 7. mRNA levels of IFN-β, interferon stimulated genes. (h) After 48 h of stimulation by irradiated tumor-supernatants, or c-di-AMP (5 ug/ml) or a combination of both, the ELISPOT assay was performed. (i) Western blots analyses of STING, p-STING, p-TBK1, p-IRF3, IRF3, and p-p65. BMDCs from cGAS-/-(k) and STING-/- (j) were co-cultured with CD8 + T cells for ELISPOT and additionally supplemented with IFN-β (10 ng/ml) and DMXAA (100 μg/ml). Representative data shown in C to E were conducted with 5 mice per group. Data are represented as mean ± SEM. *P < .05, **P < .01, ***P < .001 and ns No significant difference.

Upon sensing cytosolic DNA by cGAS, the eukaryotic cyclic dinucleotide (CDN) cGAMP is synthesized, which triggers the cGAS–STING signaling cascade. In addition to cGAMP, only bacteria can produce CDNs, including c-di-AMP, c-di-GMP, and 3′3′-cGAMP, which are directly sensed by STING.^20^ The interactions between CDNs and host immunity have been exploited in the development of bacterial vaccines.^21^ However, the importance of CDNs in RT has not yet been established. Accordingly, we evaluated the levels of c-di-AMP and c-di-GMP in fecal samples from HCC patients using liquid chromatography high-resolution mass spectrometry (LC-HRMS, n = 24). The c-di-AMP levels were significantly higher in the R group than in the NR group (*p* = .0215), Figure 5a but no significant differences were observed in the c-di-GMP levels between these groups (*p* = .4979, Figure 5b). Similarly, we found that FMT from R patients to ABX mice resulted in higher levels of c-di-AMP than those observed after FMT from NR patients (Figure 5c). The content of c-di-AMP in the ABX group was notably reduced but increased after FMT using fecal water (Figure S8a). We did not observe any variation in the c-di-AMP levels between gene-knockout and wild-type mice (Figure S8a. The administration of c-di-AMP recalled the tumor inhibitory effect of RT in the ABX and NR groups, which was likely due to the restoration of a microbiota-mediated mechanism (Figure 5d). The c-di-AMP-mediated radiosensitization was STING-dependent (Figure 5e). Therefore, we hypothesized that bacterium-derived c-di-AMP modulates RT-induced cGAS–STING signaling in DCs.

To mimic clinical settings, bone marrow DCs (BMDCs) were cultured in the presence of supernatants from irradiated tumors with or without c-di-AMP. Compared to stimulation alone, the co-stimulation group exhibited elevated type I IFN and ISG levels (Figure 5f,g). The expression levels of co-stimulatory molecules, including CD86, and chemokine receptors, including CCR1 and CCR2, were significantly higher in the co-stimulation group (Figure S8b–d). Type I IFN signaling has been reported to enhance the cross-priming of DCs, which may stimulate adaptive immune responses. We determined the effects of co-stimulation on tumor-specific T cell responses by analyzing the cross-priming ability of tumor DCs using the ELISPOT assay. Significantly elevated IFN-γ production by CD8^+^ T cells was elicited only by DCs from the group that received both c-di-AMP and irradiation (Figures 5h and S9a).

To determine whether the synergistic effects of bacterial CDNs and irradiation on DCs are mediated by the cGAS–STING–IFN-I pathway, we investigated the activation of cGAS–STING-related genes and observed elevated phosphorylation levels of STING, p65, IRF3, and TBK1 in the co-stimulation group (Figure 5i). Additionally, the induction of IFN-γ expression was eliminated in cGAS- and STING-deficient BMDCs (Figures 5j,k and S9b,c). Supplementation with exogenous IFN-β completely rescued the cross-priming impairment in both cGAS- and STING-deficient BMDCs, while supplementation with the STING agonist DMXAA exerted effects in cGAS-, but not STING-deficient BMDCs (Figures 5j,k and S9b, c).

RT leads to the extensive release of dsDNA, thereby inducing IFN-I secretion via the cGAS–STING pathway. We hypothesized that dsDNA would act synergistically with bacterial c-di-AMP to regulate the sensitivity to RT through the cGAS–STING–IFN-I pathway. We found an accumulation of cytosolic dsDNA in liver cancer cells after exposure to radiation (Figure S10a). To validate our hypothesis *in vitro*, we used poly (dA:dT) – a synthetic analog of dsDNA. Consistent with the *in vivo* results, the levels of IFN-β and Oasl2 were elevated in the co-stimulation group, whereas the synergistic elevation disappeared in cGAS- and STING-deficient cells (Figure S10b). These findings indicate that the synergistic stimulation of c-di-AMP from the gut microbiome and dsDNA from irradiated tumor cells was cGAS–STING–IFN-I signaling-dependent.

## Discussion

**Figure 6.**
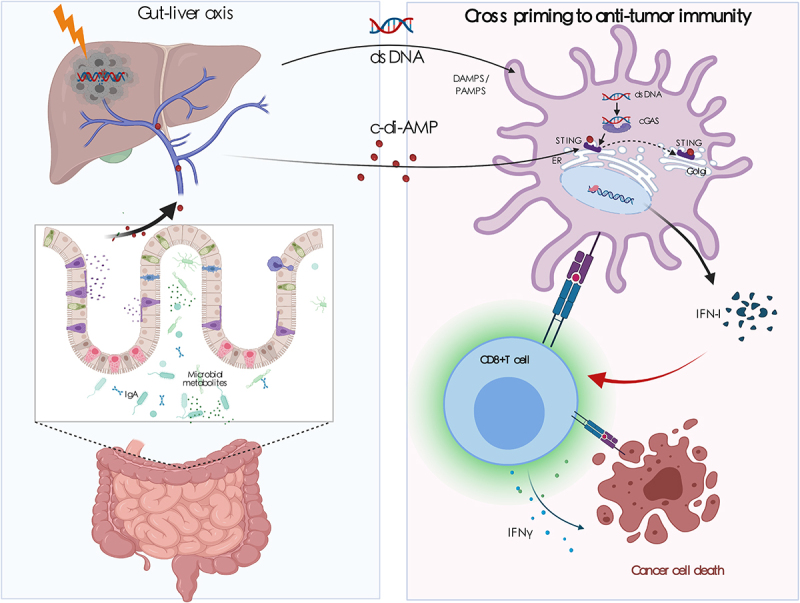
***Gut microbiota modulate radiotherapy-based*** ***antitumor immune responses against hepatocellular carcinoma through STING signaling.*** As the key frontline immune organ, the liver is constantly exposed to gut-derived signals (bacterial products, food antigens, and environmental toxins) through the biliary tract, portal vein, and systemic circulation. Based on the liver–gut axis, we showed that the disruption of the gut microbiome impairs radiotherapeutic efficacy in HCC by suppressing antigen presentation and inhibiting effector T cell functions through the cGAS/STING/I-IFN pathway. Furthermore, we identified that the bacterial second messenger c-di-AMP was higher in the R group and may be an important mediator through which the gut microbiome exerts its immune regulatory effects. C-di-AMP can synergize with radiotherapy (dsDNA) to promote the maturation and presentation functions of dendritic cells, strengthening IFN-β production and CD8+ cytolytic T cell activation in a cGAS- and STING-dependent manner. Our research provides evidence for the previously unexplored role of the gut microbiome in the regulation of RT in HCC and establishes a crucial link between bacterial c-di-AMP and the host cGAS/STING pathway via the gut microbiome–liver axis.

Our understanding of host–microbial interactions as part of the mammalian holobiont has increased substantially over the past two decades.^22^ Growing evidence has proven that alterations in the composition of gut microbiota, or dysbacteriosis, are associated with the occurrence and development of various diseases, including inflammatory, metabolic, and autoimmune diseases, as well as cancers.^23^ Changes in the gut microbiome can also modify the efficacy and toxicity of cancer therapies.^24^ Understanding the mechanisms through which microorganisms interact with the host may yield more effective treatment strategies for various diseases. In the present study, we elucidated the previously unexplored role of the gut microbiome in the regulation of HCC patient response to RT and described a crucial link between bacterial c-di-AMP and the host cGAS–STING pathway via the gut microbiome–liver axis (Figure 6).

The unique double-blood supply structure in the liver allows it to continuously receive external antigen stimulation from the portal vein. While defending against external antigens, immune molecules in the liver also form a microenvironment of autoimmune tolerance; in this scenario, high levels of PD-L1 and low levels of CD80 and CD86 are expressed, resulting in the underactivation of CD4^+^ and CD8^+^ T cells.^25–27^ Most HCCs develop from chronic liver diseases and present with serious dysbiosis, which may further aggravate the immunosuppressive microenvironment.^28^ Stereotactic body RT (SBRT) is an effective and safe local treatment for HCC.^29^ The traditional view is that RT directly kills tumor cells, while the current perception is that RT can also cause tumor cells to release “danger signals,” causing a secondary response and exerting antitumor effects by activating the immune system.^30,31^ Indeed, the immune system plays a particularly important role in RT for HCC.^32^ Given the importance of gut microbiota in modulating the host immune response, we hypothesized that the gut microbiome may be a critical immunomodulator during RT. Patients with a healthy gut microbiome (high abundance of members from Clostridiales, *Ruminococcaceae*, and *Faecalibacterium*) exhibited stronger effector T cell functions in the TME. In contrast, patients presenting with gut dysbacteriosis (high abundance of members from Lactobacillales and *Streptococcus*) exhibited impaired antitumor immune responses. We propose that bacterial c-di-AMP and dsDNA synergistically trigger IFN-β production and CD8^+^ cytolytic T cell activation in a STING-dependent manner. Although only 24 patients were enrolled in our study, our results clearly emphasize the immunomodulatory role of the gut microbiome and provide a potential strategy to promote the sensitization to RT in HCC.

The key mediators of communication between the host and microorganisms are PRRs, which are expressed by innate immune cells, such as DCs, monocytes/macrophages, and natural killer cells. Upon the recognition of a pathogen, IFN induction is rapidly triggered.^5,33^ Laura *et al*. showed that the induction of the expression of type I IFN by gut microbiota maintains DCs in a poised basal state, enabling a robust response to pathogens. In the absence of stimulation with microbiota, DCs cannot appropriately participate in the immune activation process, resulting in higher sensitivity to various viral infections and reduced tumor suppression.^34^ STING-an essential signal adaptor of the cytosolic surveillance system-acts as the converging point for several recently identified DNA sensors (cGAS, Mre11, IFI16, DDX41, and DNA-PKcs).^35^ Emerging studies have shown that the cGAS–STING pathway plays an essential role in HCC progression. Additionally, low levels of STING in tumor tissues have been reported to be associated with exhausted inflammatory infiltration and poor prognosis in patients with HCC. Hepatocytes do not contain functional STING; therefore, the antitumor effects of the cGAS–STING pathway come from non-parenchymal liver cells. Treatment with DMXAA, a STING agonist, has failed in human clinical trials, likely because it is unable to stimulate hSTING. Our findings elucidate the regulatory effects of the gut microbiome on the immune microenvironment after RT for HCC and suggest that a healthy gut microbiome may have clinical value as an extracellular agonist of STING, which plays essential roles in gut microbiome-mediated antitumor immune modulation.

Most studies of the association between the gut microbiome and tumor treatment have focused on metabolic factors, including short chain fatty acids and butyrate, but have not assessed the impact of bacterial metabolites.^6,36,37^ c-di-AMP (mostly synthesized by gram-positive bacteria) and c-di-GMP (mostly synthesized by gram-negative bacteria) are important second messenger molecules; they are involved in stimulating immune responses in eukaryotic hosts and have been utilized as vaccine adjuvants for influenza and hepatitis C.^21,38^ We found a higher level of c-di-AMP in the R group, and c-di-AMP cooperated with dsDNA to promote DC maturation and type I IFN release. In our analysis of microbe species abundance, we found that *Faecalibacterium* (gram-positive bacteria) was enriched in the R group and *Streptococcus* was enriched in the NR group. Warrison *et al*. previously reported that IFN-β production induced by members of group B *Streptococcus* is almost exclusively dependent on the cGAS–STING pathway. Members of group B *Streptococcus* produce the ectonucleotidase CdnP, which hydrolyzes extracellular bacterial c-di-AMP. The enrichment of group B *Streptococcus* leads to the suppression of IFN-β production via the degradation of c-di-AMP and the inhibition of STING overactivation.^39^ The types of bacteria responsible for the higher levels of c-di-AMP in the R group need to be further verified by performing the transplantation of single, specific bacterial strains.

Bacterial c-di-AMP and c-di-GMP can be directly recognized by STING to trigger downstream immune-associated kinases, leading to the secretion of type I IFNs and proinflammatory cytokines.^40^ The combination of c-di-AMP and c-di-GMP enhances the formation of STING dimers to trigger stronger IFN production.^38^ Liu *et al*. reported that extracellular CDNs (eCDNs; c-di-AMP and c-di-GMP) differ substantially from intracellular CDNs (cGAMP). STING is important but insufficient for eCDN-induced type I production; thus, eCDNs require cGAS for STING activation. cGAS serves as a scaffolding protein and nucleates the formation of perinuclear signalosomes encompassing eCDNs/cGAS/STING, thereby enabling STING activation. The binding of eCDNs promotes their interaction with cGAS and STING, which is important for the recruitment of STING to the perinuclear region.^41^ Our *in vitro* and *in vivo* results demonstrate that c-di-AMP can synergistically enhance the antitumor effects of RT-associated dsDNA by promoting IFN-β production and CD8^+^ cytolytic T cell activation in a cGAS- and STING-dependent manner. Our study has further highlighted the role of c-di-AMP as a predictor of radiosensitivity in HCC. In addition, we propose the potential application value of c-di-AMP in targeting the cGAS/STING pathway for radiosensitization. C-di-AMP may therefore serve as a unique bridge between the gut microbiome and regulation of RT efficacy in HCC.

To the best of our knowledge, this is the first study describing a potential relationship between the gut microbiome and sensitivity to RT in liver cancer. Furthermore, our study provides a potential approach for modulating and predicting the efficacy of RT through the gut microbiome–liver axis.

## Materials and methods

### Patient recruitment and fecal collection

This prospective study was approved by the Institutional Review Board of Zhongshan Hospital, Fudan University. Written informed consents were obtained from all participants before enrollment. The inclusion criteria were: 1) Patients with clinically diagnosed hepatocellular carcinoma (HCC) according to American Association for the Study of Liver Diseases Guidelines;^42^ 2) Participants with unresectable intrahepatic tumors, treated with external radiation (50–60 Gy, 2.0–3.0 Gy/f) and who had adequate follow-up information; 3) Patients whose fecal samples were collected before RT. Patients were excluded if they had been previously exposed to any antibiotics, prebiotics, probiotics, steroids, or immune-suppressants within four weeks prior to fecal sampling. In addition, fecal samples from healthy volunteers were obtained as the control group.

### Clinical information and treatment response evaluation

Demographic, cancer-related and treatment information were collected. RT strategy was as previously described.^43^ The general characteristics of the patients are shown in Table 1. Contrast-enhancement magnetic resonance images were performed 1.5–2.0 months after completion of external RT. Treatment responses were evaluated by independent radiologists, according to the revised Response Evaluation Criteria in Solid Tumors (version 1.1).^44^ HCC with complete remission (CR) or partial remission (PR) were classified as the response group (R), while patients with stable disease (SD) or progress disease (PD) were defined as the no response group (NR).

### Patient fecal microbiota transplantation and Administration of c-di-AMP

Mice were pretreated with a three-antibiotic cocktail for 3 days. Fecal samples from three responder (R) or three non-responder (NR) HCC donors were individually transferred into independent of 4 ABX mice per donor. fecal samples were resuspended into sterile 200 uL PBS for 0.1 g/ml. Fecal suspension was obtained using a 100 mm strainer and gavaged into ABX pre-treated recipients for 3 doses over 1 week, followed by a 1-week break to allow for the establishment of the microbiota. For in vivo administration of c-di-AMP (InvivoGen), mice were injected intraperitoneally with 25ug one day before radiotherapy, and once every other day, a total of three times.

### Cell culture and reagents

Human HCC cell lines HCCLM3 and mouse HCC cell lines Hepa1-6 were obtained from the Cell Bank of Chinese Academy of Sciences, and both were cultured in H-DMEM containing 10% fetal bovine serum, and penicillin/streptomycin. Mouse HCC cell lines H22 were obtained from the Cell Bank of Chinese Academy of Sciences and cultured in RPMI 1640 containing 10% fetal bovine serum, and penicillin/streptomycin. The H22-OVA cells were selected for a single clone after H22 being transduced by lentivirus expressing Luciferin and Ovalbumin. DMXAA and c-di-AMP were purchased from MCE. Murine IFN‐β, IL-4 and GM‐CSF was purchased from PEPROTECH.

### Murine studies

C57BL/6 male mice and cGAS KO (Mb21d1^–/–^) mice were obtained from The Jackson Laboratory (USA). STING-deficient mice (Tmem173gt) were kindly provided by Dr. Liufu Deng (Shanghai Jiaotong University, China). Mice were maintained in a specific pathogen-free environment (all water and food were autoclaved to protect against pathogenic contamination). Upon the new arrivals, mice were housed for one week to normalize gut microbiome and randomly divided into 4 groups. As previously reported, ABX treatment groups were treated with a three-antibiotic cocktail (vancomycin (500 mg/L), imipenem/cilastatin (500 mg/L) and neomycin (1 g/L)) in drinking water, which was changed every two days.^45^ After 3 weeks of ABX pretreatment, mice were subjected to the tumor bearing experiment. Murine HCC H22-OVA cells were injected into the right flanks of mice. When tumor volumes reached ~80 mm3, mice were exposed to 8 Gy radiation for 3 consecutive days. For fecal microbial transplantation experiments, mice were pretreated with a three-antibiotic cocktail for 3 days. With reference to previous studies, fecal samples from Water or ABX groups were resuspended in sterile PBS and gavaged into ABX pre-treated recipients.^46^ In addition, coating the fur with feces suspension helps in better assimilation. Two weeks after FMT, H22-OVA cells were subcutaneously injected in mice, after which mice were treated as mentioned above. For the CD8 depletion assay, anti-mouse monoclonal CD8-blocking antibody or isotype control mAb were intraperitoneally injected every three days starting on day one before radiation at a dose of 200 µg per mouse.

### 16S rRNA sequencing and analysis

The conserved V3-V4 region in the 16S rDNA sequence was amplified by the Phusion enzyme, after which the sequencing universal connector and sample specific barcode sequences were added to the amplification product of the target region by PCR. PCR products were detected by 1.5% agarose gel electrophoresis. Target fragments were retrieved and purified. Purified PCR products were quantified and mixed with the library using the quantum it PicoGreen dsDNA assay kit on the Promega quantifluor fluorescence quantitative system. Finally, we used the Illumina sequencing platform, 250PE, for double ended sequencing according to standard procedures.

The original off-line data obtained by sequencing was spliced by overlap, after which quality control and chimera filtering was performed to obtain high-quality clean data. DADA2 (Divisive Amplicon Denoising Algorithm) no longer clusters by sequence similarity, but obtains representative sequences with single base accuracy through steps such as “de duplication”, which improves data accuracy and species resolution. The core of dada2 is denoising. The final feature table and feature sequence are obtained through qiime2 for further diversity analysis, species classification annotation and difference analysis.

### ELISA assay

IFN-γ levels were measured using a mouse IFN-γ ELISA kit (Elabscience), as described by the manufacturer.

### Flow cytometry analysis

Tumors were harvested and dissociated into single-cell suspensions. Then, cells were blocked with anti‐FcR (clone 2.4 G2, BD Pharmingen) and labeled with indicated surface markers for 30 min at 4°C. For IFN-γ staining, single-cells were cultured in the presence of a cell activation cocktail (with Brefeldin A) (Biolegend) for 5 h. Cells were permeabilized and stained with intracellular antibodies for 30 min at 4°C as instructed by the manufacturer. Dead cells were excluded using LIVE/DEAD Fixable Dead Cell Stain Kit (Invitrogen). The antibodies used in the flow cytometry analysis were: anti-CD45 (clone 30-F11, Invitrogen), anti-CD3 (clone 145–2C11, Biolegend), anti CD4 (clone RM4-5, Biolegend), anti-CD8 (clone 53–6.7, Biolegend), anti-IFN-γ (clone XMG1.2, Biolegend), anti-CD11C (clone N418, Biolegend), anti-CD80 (clone 16–10A1, Biolegend), and anti-CD86 (clone GL1, Invitrogen). Because the specific reaction with ovalbumin-derived peptide SIINFEKL bound to H-2 Kb of MHC class I, anti–H-2kb bound to SIINFEKL (clone 25-D1.16, Biolegend) was used to recognize the tumor specific immune cells. Flow cytometry was performed on the FACS Aria III platform (BD Biosciences, San Jose, CA, USA) and results analyzed using FlowJo software version 10.4 (TreeStar).

### Preparation of bone marrow- derived DC (BMDC)

Bone marrow cells were flushed out form the mouse femur and add into red blood cell lysis buffer. The cells were washed and cultured in RPMI-1640 medium containing 10% FBS, supplemented with 20 ng/ml mGM-CSF and 20 ng/ml mIL-4. Fresh media with supplements were replaced every 2 days. On day 7, cells were stimulated with irradiated tumor-supernatant, c-di-AMP or LPS (Beyotime Biotechnology) overnight according to the grouping.

### IFN-γ ELISPOT

CD11c+ dendritic cells were sorted using FACSAriaIII (BD) or mouse CD11c positive selection kit (Biolegend). After grinding and lysis of the red blood cells in the spleen, CD8+ positive T cells were sorted using a mouse CD8+ naïve T cell isolation kit (Biolegend), according to the manufacturer’s instructions. CD11c+ cells isolated from the TDLN cell suspension or BMDC were co-cultured with purified naive CD8 + T cells at a ratio of 1:10 followed by overnight incubation in the presence of OVA peptide (257–264). For the rescue assay, murine IFN-β (10 ng/ml) or DMXAA (100 μg/ml) were added in the co-culture of DCs and CD8 + T cells. Spots of IFN-γ were detected by mouse IFN-γ precoated ELISPOT kit, according to the manufacturer’s instructions (DaYou, 2210005). Spots were recognized by an automated ELISPOT reader (Mabtech IRIS FluoroSpot/ELISpot) using the RAWspot technology for multiplexing at the single-cell level.

### RNA extraction and quantitative real-time RT-PCR

Total RNA was extracted using TRIzol reagent (Invitrogen). 1000 nanograms of total RNA was reverse transcribed into cDNA according to the manufacturer’s instructions (Vazyme). Real-time quantitative RT-PCR was then performed using the Hieff UNICON® Power qPCR SYBR Green Master Mix (Yeasen) with forward and reverse primers at a final concentration of 0.3 µM, in a sample volume of 10 µL. The sequences of primers are listed in Supplemental Table. Data were normalized by the level of beta actin. The 2-ΔΔCt method was used to calculate the relative expression changes.

### Histology and immunohistochemistry

Fresh tumor tissues were fixed in 4% paraformaldehyde and embedded in paraffin, then cut into 5 μm sections. Follows by dewaxing, rehydration, antigen repair, blocking, the slides were incubated with the primary antibodies overnight. On day 2, the slides were incubated with the HRP-labeled or fluorescent conjugated second antibody, developed with DAB and counterstained with hematoxylin. DAPI was used for nuclear staining in immunofluorescence. TUNEL staining was performed using a One-step TUNEL Assay Kit (Green, FITC) E-CK-A320 (Elabscience) to detect apoptosis of the tumors according to the manufacturer’s instructions. Images were captured by microscope and analyzed using Image J software (NIH).

### RNA-seq

Total RNA was extracted from samples, purified, and their concentrations and purity determined by nanodrop nd-1000 (NanoDrop, Wilmington, DE, USA). The integrity of RNA was detected by Bioanalyzer 2100 (Agilent, CA, USA). Concentration >50 ng/μL, Rin value > 7.0, OD260/280 > 1.8, total RNA >1 μg met the downstream test. Oligo (DT) magnetic beads (Dynabeads, oligo (DT), Thermo Fisher, USA) were used to specifically capture mRNA with poly A. Captured mRNA was fragmented (NEBNext® Magnesium RNA Fragmentation Module, USA). Fragmented RNA was reversed to synthesize cDNA. Then, E. coli DNA polymerase I (NEB, USA) and RNase H (NEB, USA) were used for two strand synthesis. These DNA and RNA were transformed into DNA double strands. At the same time, the dUTP solution (Thermo Fisher, CA, USA) was added into the ends of the double stranded DNA to fill the flat ends. Then, an A deoxynucleotide was added at both ends to connect it with the connector with T deoxynucleotides at the end. The fragment was retrieved and purified by magnetic beads. The second strand was digested using the UDG enzyme (NEB, Ma, US), after which a library with fragment size of 300 bp ± 50 bp was formed by PCR. Finally, Illumina Novaseq™ 6000 (LC Bio Technology CO., Ltd. Hangzhou, China) was used to perform pair-end sequencing according to standard operations. The sequencing mode was PE150.

The Cutadapt software was used to remove reads that contained adaptor contamination. After removal of low-quality bases and undetermined bases, the HISAT2 software was used to map reads to the genome. Mapped reads for each sample were assembled using StringTie with default parameters. Then, all transcriptomes from all samples were merged to reconstruct a comprehensive transcriptome using the gffcompare software. After the generation of the final transcriptome, StringTie and ballgown were used to estimate the expression levels of all transcripts and to evaluate the expression levels of mRNAs by calculating FPKM. The edgeR R package was used to analyze significant differences between samples. Genes with difference factors FC>2 or FC<0.5 and p < .05 were defined as differential genes, after which GO and KEGG enrichment analyses were performed.

### Ultraperformance liquid chromatography/mass spectrometry analysis

Stools and fecal samples were collected and stored frozen in sterile tubes until analysis. The samples were slowly thawed on ice, weigh the appropriate into 2 ml centrifuge tubes. Then, 500 μ L 80% methanol was added for grinding and ultrasound. After centrifugation, take 15 μ l of the supernatant was used for detection. Instrument: liquid chromatography was performed by the Waters Acquisition UPLC while mass spectrometry was performed by AB SCIEX 5500 QQQ -MS.

Chromatographic column: Acquity UPLC HSS T3. Mobile phase: A, water; B, acetonitrile. MS parameters were: Curtain Gas, 20 arb; IonSpray voltage, −4500 V; Temperature, 450°C; IonSource Gas1, 55 arb; IonSource Gas2, 55 arb. Concentrations of c-di-AMP were determined as: y = 797.49x – 552.32; R2 = 0.9995. Concentrations of c-di-GMP were determined as: y = 703.59x – 1081.8; R2 = 0.9996 (y, peak area; x, analyte concentration in ng/ml). Standards for c-di-AMP, c-di-GMP were purchased from MCE.

### Statistics

The sample sizes were guided by the basis of pilot experiments and the previous studies in our laboratory. Statistical analysis was conducted by GraphPad Prism 7 (GraphPad Software). The significant differences between groups were calculated by Student’s unpaired t-test, one-way, or two-way ANOVA (Tukey’s and Bonferroni’s multiple comparison test). P < .05 was considered significant.

## Supplementary Material

Supplemental Material

## Data Availability

The data that support the findings of this study are openly available in the BioProject database at https://dataview.ncbi.nlm.nih.gov/object/PRJNA825325, reference number PRJNA825325.
