# Supplementary material for: Gut microbiota modulate radiotherapy-associated antitumor immune responses against hepatocellular carcinoma Via STING signaling
Source: Gut Microbes. 2022 Sep 10;14(1):2119055. doi: 10.1080/19490976.2022.2119055 (PMC9467592; doi:10.1080/19490976.2022.2119055)
Supplement: Supplemental Material [file KGMI_A_2119055_SM8919.docx]

**
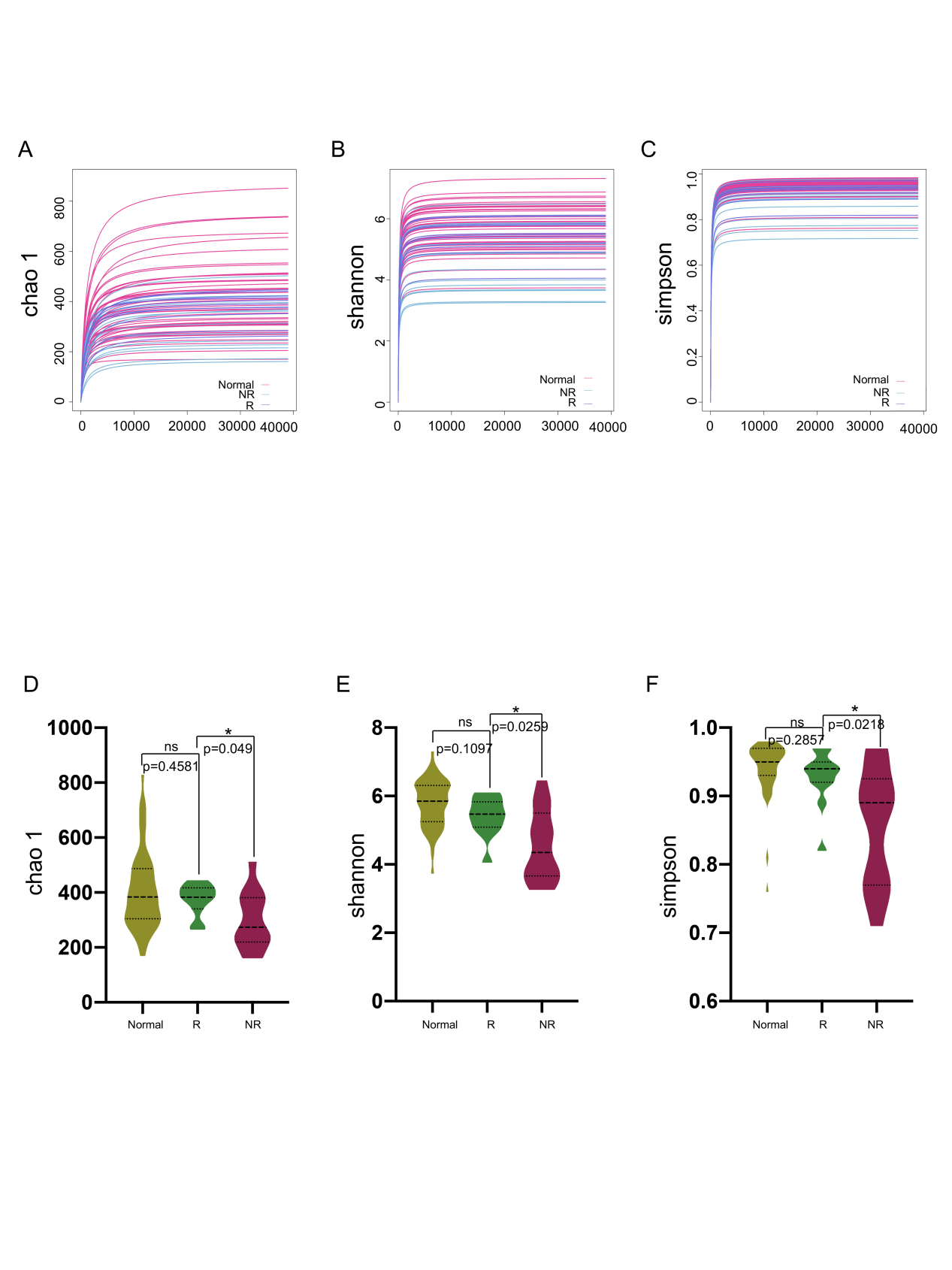
**

**Supplementary Figure 1: Alpha diversity of gut microbiome in liver cancer patients** (A-C)The rarefaction analysis between the number of sequence and the

number of OTUs; Comparisons of alpha diversity scores in healthy individuals (n=46), R group (n= 11)and NR group (n=13) using the (D) Chao1 index, (E) Shannon index, and (F) simpson index by Wilcoxon test. * p<0.05, ** p<0.01.


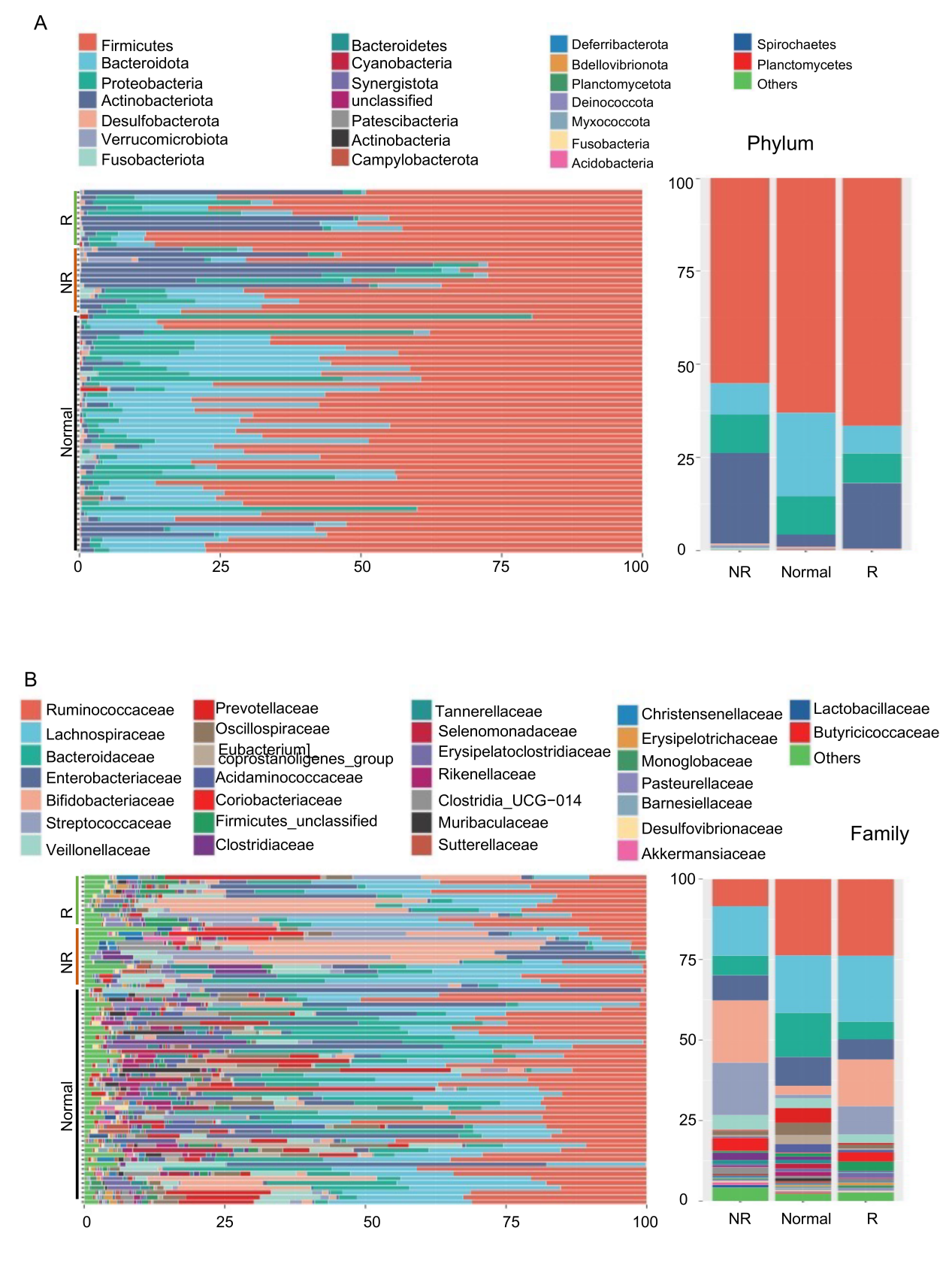


**Supplementary Figure 2: Gut microbiota composition of HCC patients were determined by 16S rRNA gene sequencing** Stacked bar chart shows phylum levels (A)of microbial composition in individual or groups; and family levels (B) of microbial compositions in individuals or groups.


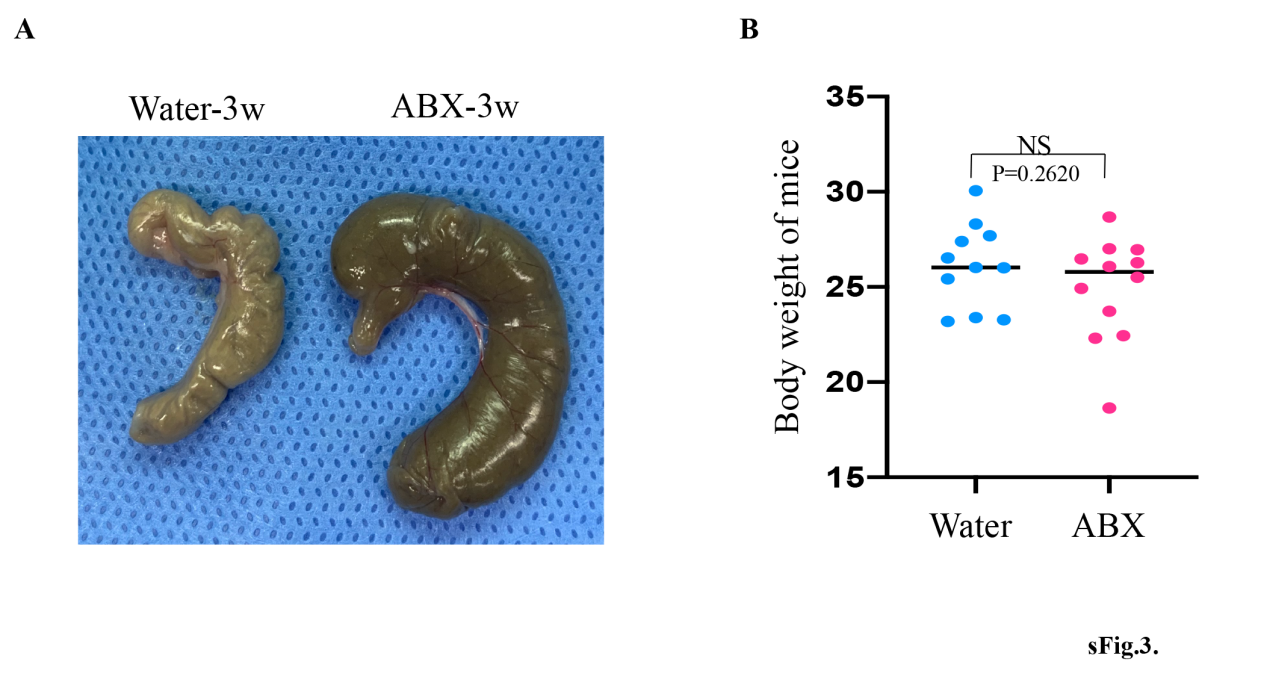


**Supplementary Figure 3: Construction of the dysbacteriosis model using** **an antibiotic cocktail** (A) Images of the cecum after Water or ABX treatment. (B) Body weights of mice after Water or ABX treatment.


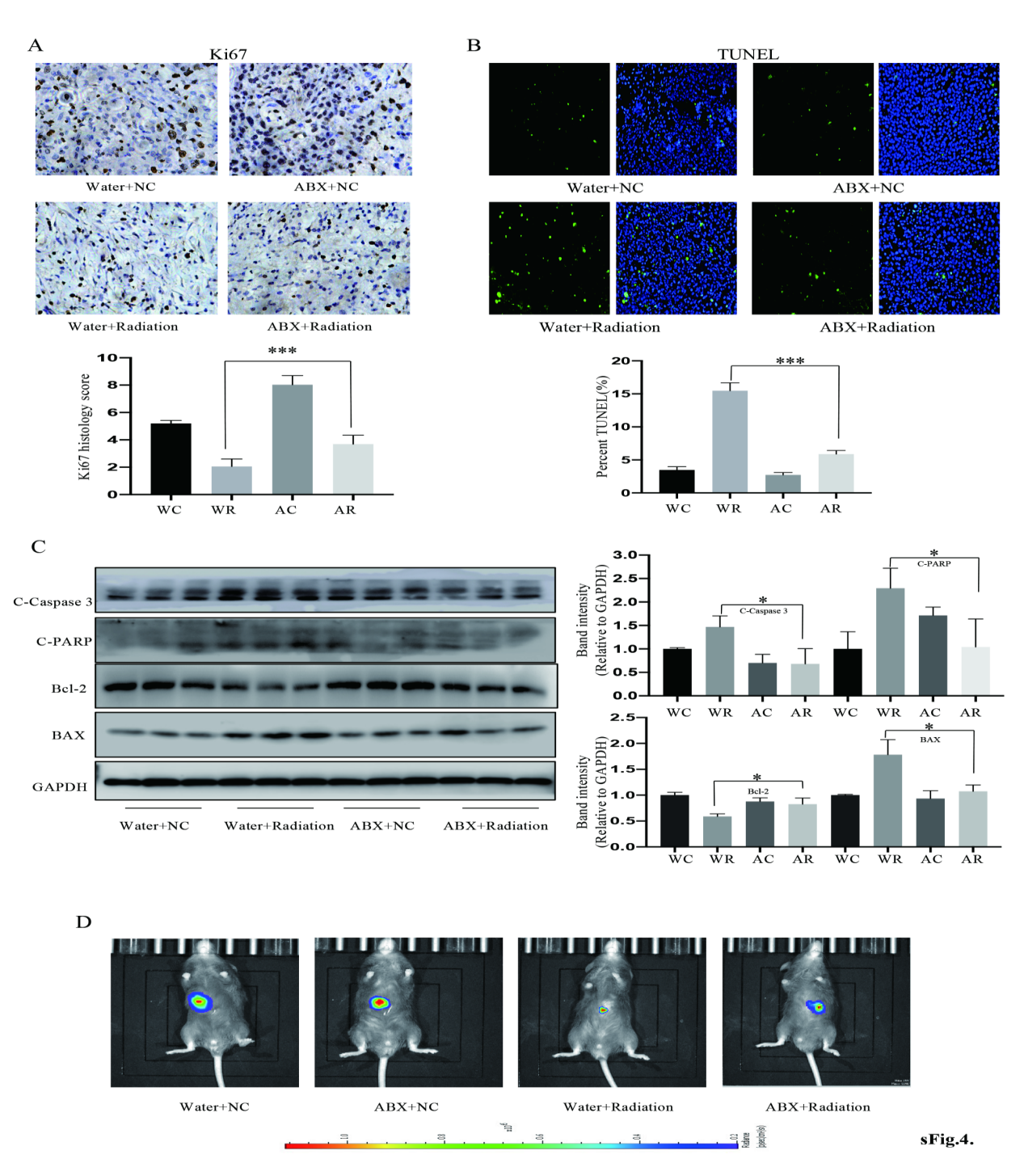


**Supplementary Figure 4: Dysbiosis of gut microbiome inhibited radiation induced-apoptosis, impairing the efficacy of radiotherapy in mouse HCC tumor models** Representative images and immunohistochemical quantification of Ki67 staining (A) and TUNEL (B) in Water or ABX treated mice after radiotherapy. (C) Western blot analysis of Cleave-Caspase3, Cleave-PARP, Bcl-2, BAX in the Water or ABX treatment mice after radiotherapy. (D) In vivo imaging of liver tumor in situ.


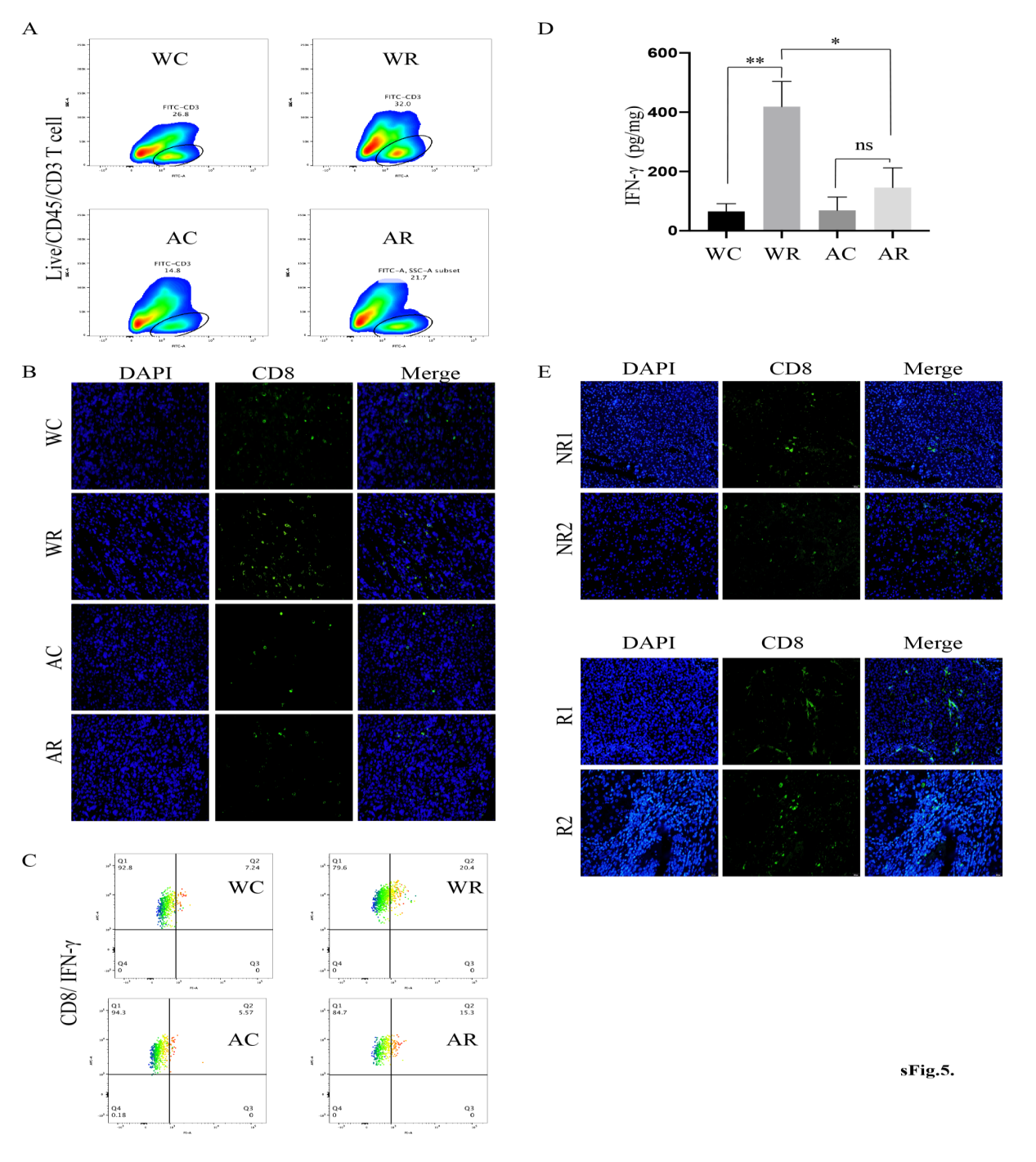


**Supplementary Figure 5:** **Crucial roles of cytolytic CD8+ T cells in gut microbiome mediated antitumor immunity regulation during HCC radiotherapy**

1. Representative images of tumor-infiltrating CD3+ T cell populations (Live/CD45+/CD3+cells), by flow cytometry. (B) Representative images of tumor infiltrating CD8+ T cells in paraffin sections of mice models. (C) Representative images of tumor-infiltrating CD8/IFN-γ + population (Live/CD45/CD3/CD8/IFN-γ cells) by flow cytometry. (D) Concentrations of IFN-γ in tumor tissues were measured by ELISA, and values expressed as pg/mg. (E) Representative images of tumor infiltrating CD8+ T cells in paraffin sections of HCC patients.


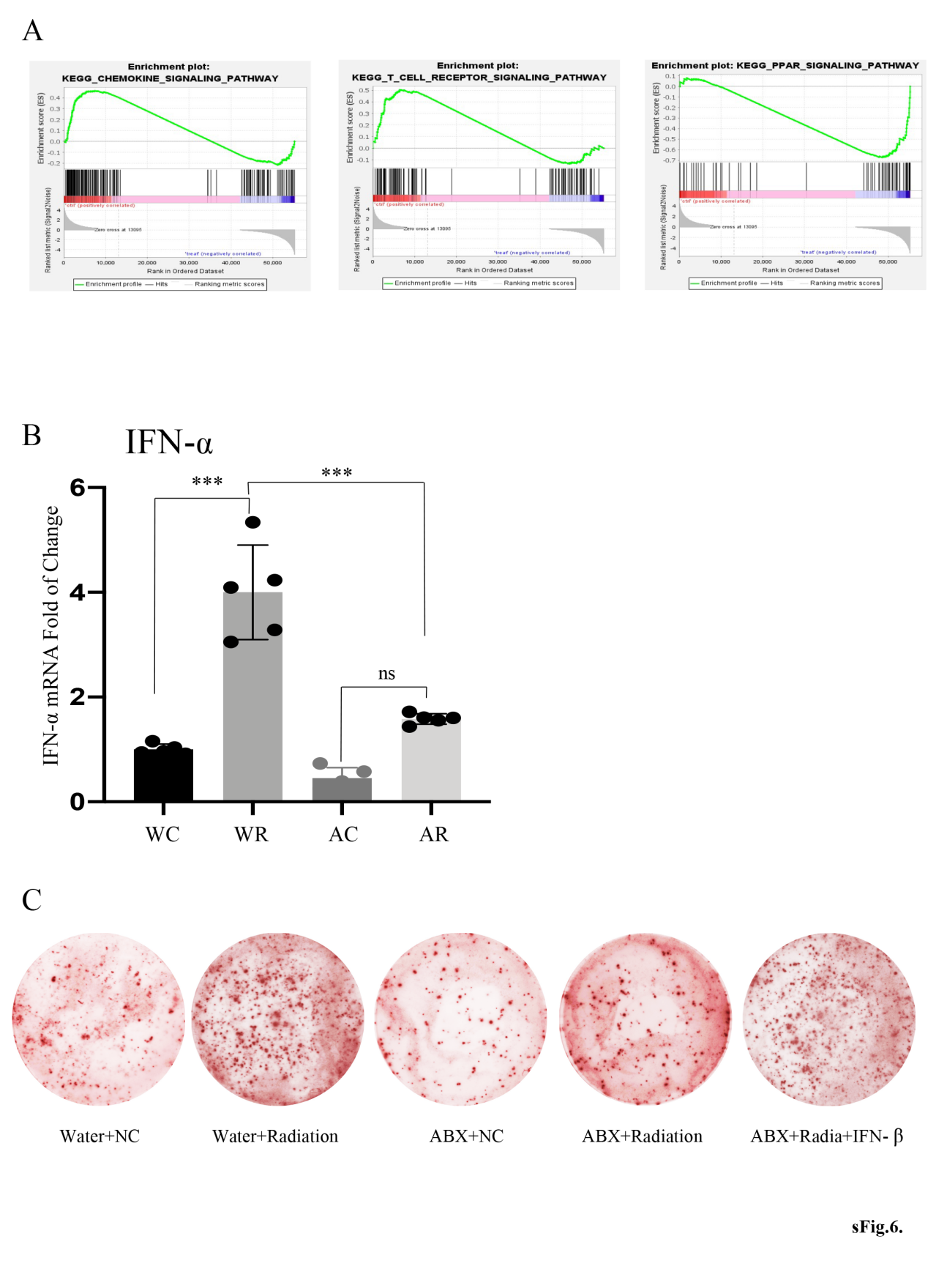


**Supplementary Figure 6: ABX-treatment suppressed the ability of dendritic cells for antigen presentation and T cell activation** (A) GSEA analysis of Chemokine, T cell receptor and PPAR signaling pathways. (B) Quantification of IFN-α mRNA levels in TDLN by qRT-PCR. (C) Representative images of the IFN-γ ELISPOT assay.


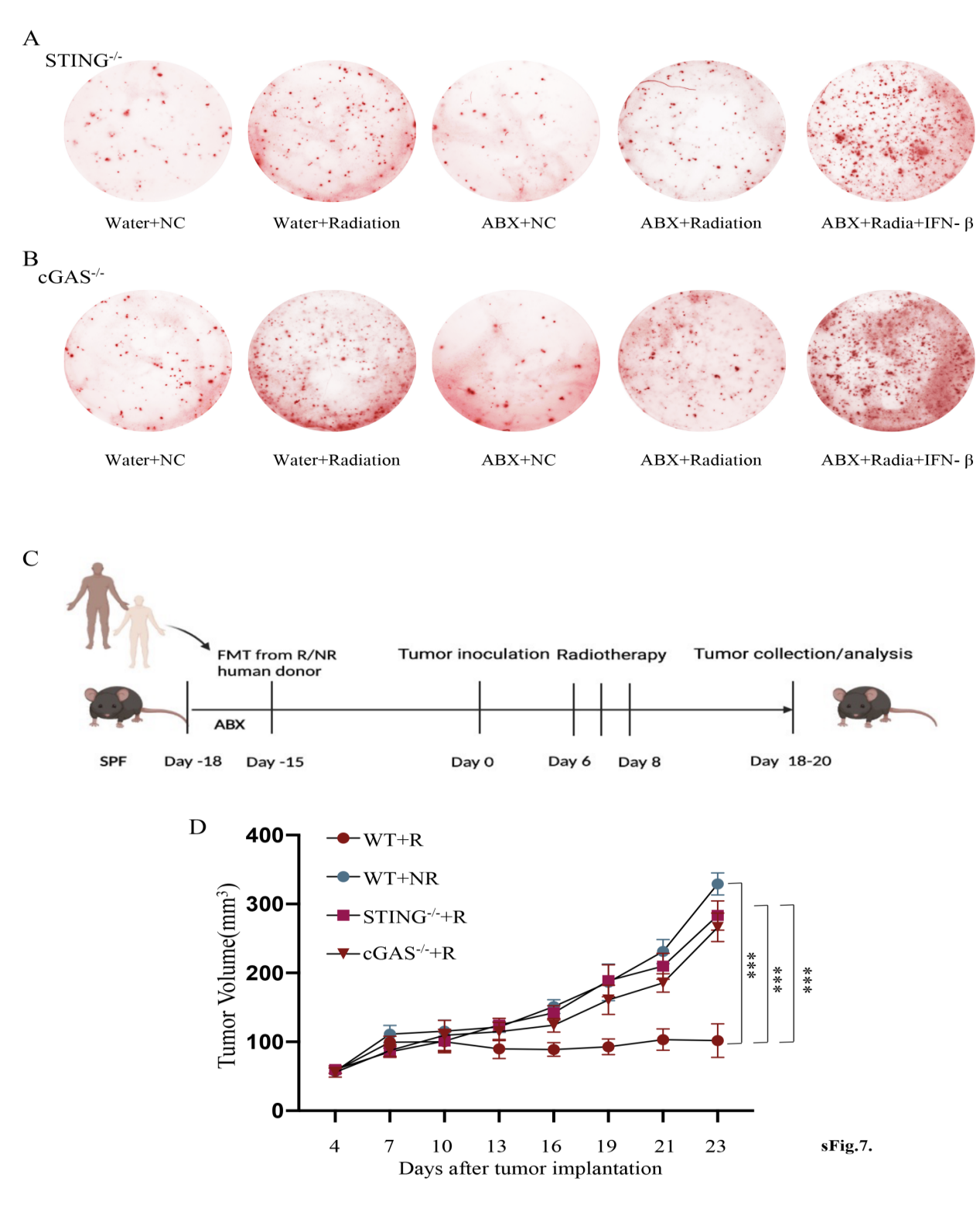


**Supplementary** **Figure 7: cGAS/STING/****IFN-I pathway plays a vital role in gut microbiome mediated antitumor immune regulation during HCC radiotherapy**

Purified CD11c+ DC cells from (A) STING-/- and (B) cGAS-/- mice models were co-cultured with naive CD8+ T cells with or without IFN-β (10 ng/ml), and IFN-γ secretion was detected by ELISPOT assay. Representative images of IFN-γ ELISPOT assay are shown. (C) The pattern diagram of patient fecal microbiota transplantation. (D) Representative H22-OVA tumor growth curves after fecal microbiota transplantation in the wild type, STING-/- and cGAS-/- mice.


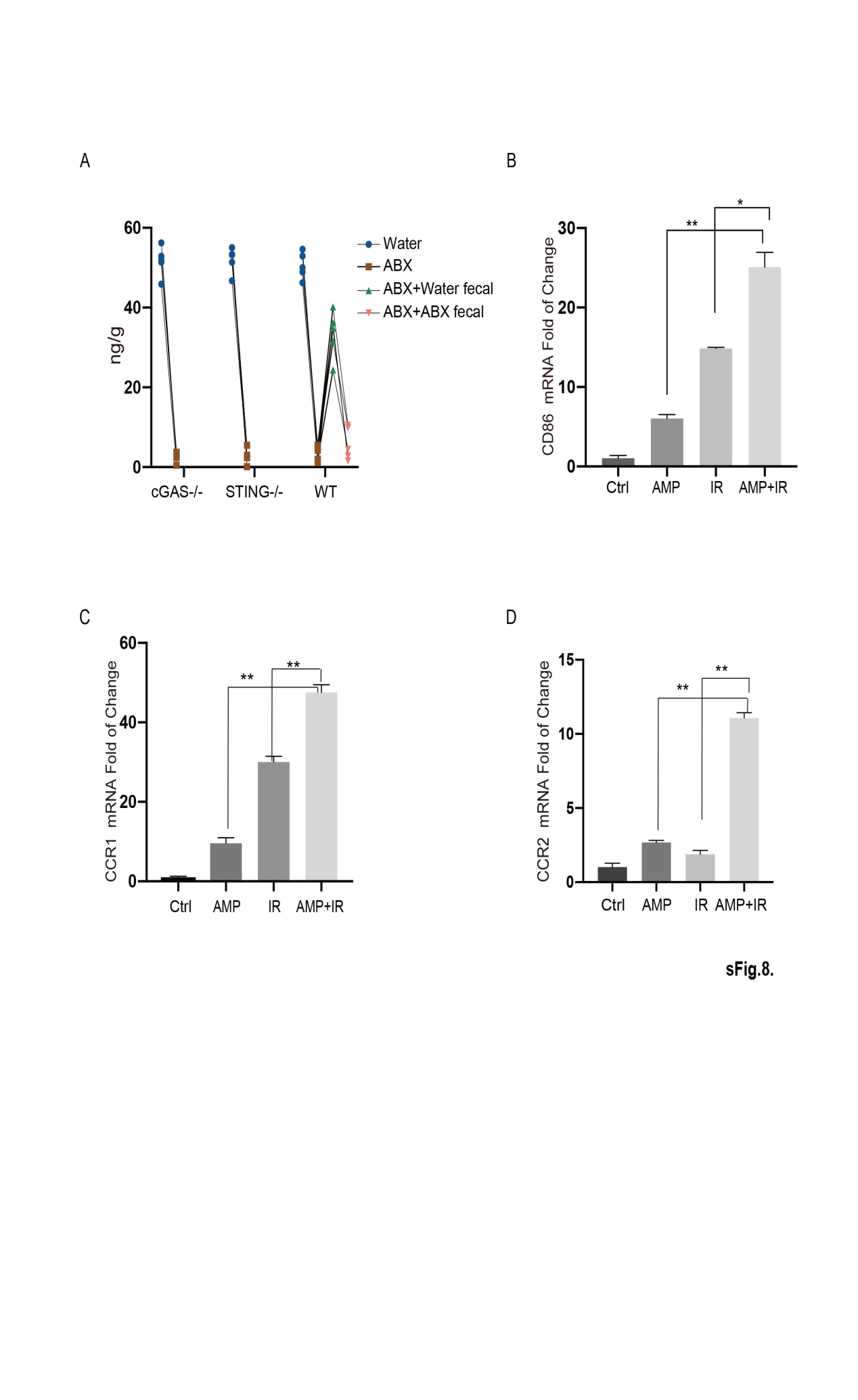


**Supplementary Figure 8: Combination of c-di-AMP with radiation promotes the maturation and activation of dendritic cells** (A) After FMT with water or ABX fecal, the content of c-di-AMP was detected by UPLC/MS in the wild type, STING-/- and cGAS-/- mice. (B-D) On day 7, BMDCs were stimulated with irradiated tumor-supernatants, or c-di-AMP (5 ug/ml) or a combination for 48 h. mRNA levels of CD86, CCR1 and CCR2 were detected by qPCR.


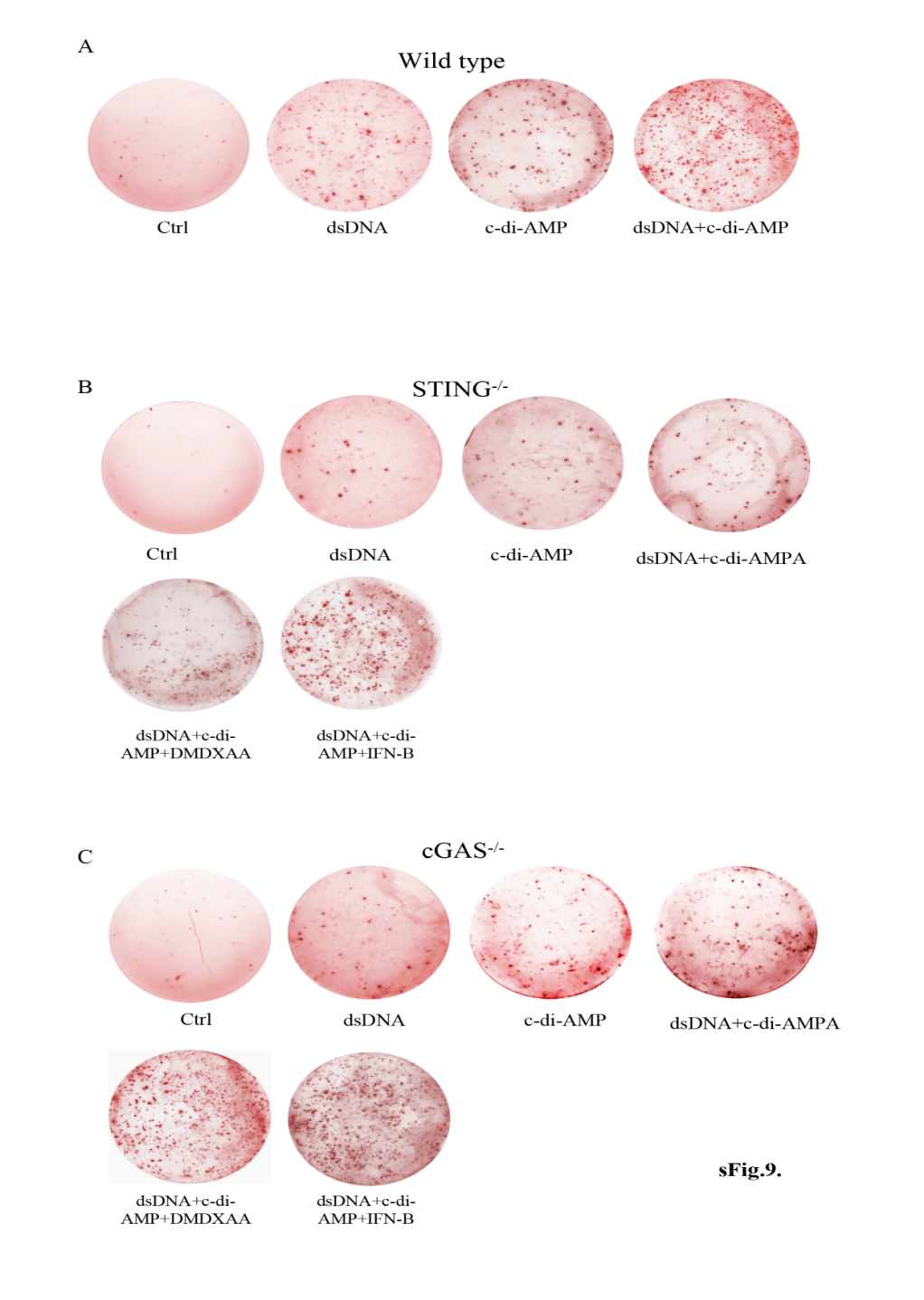


**Supplementary Figure 9:** **Combination of c-di-AMP with radiation promotes IFN-β and IFNγ secretion** BMDCs were stimulated with irradiated tumor-supernatants, or c-di-AMP (5 ug/ml) or their combination for 48 h. Representative images of IFN-γ ELISPOT assay in wild type (A), cGAS-/-(C) and STING-/- (B).


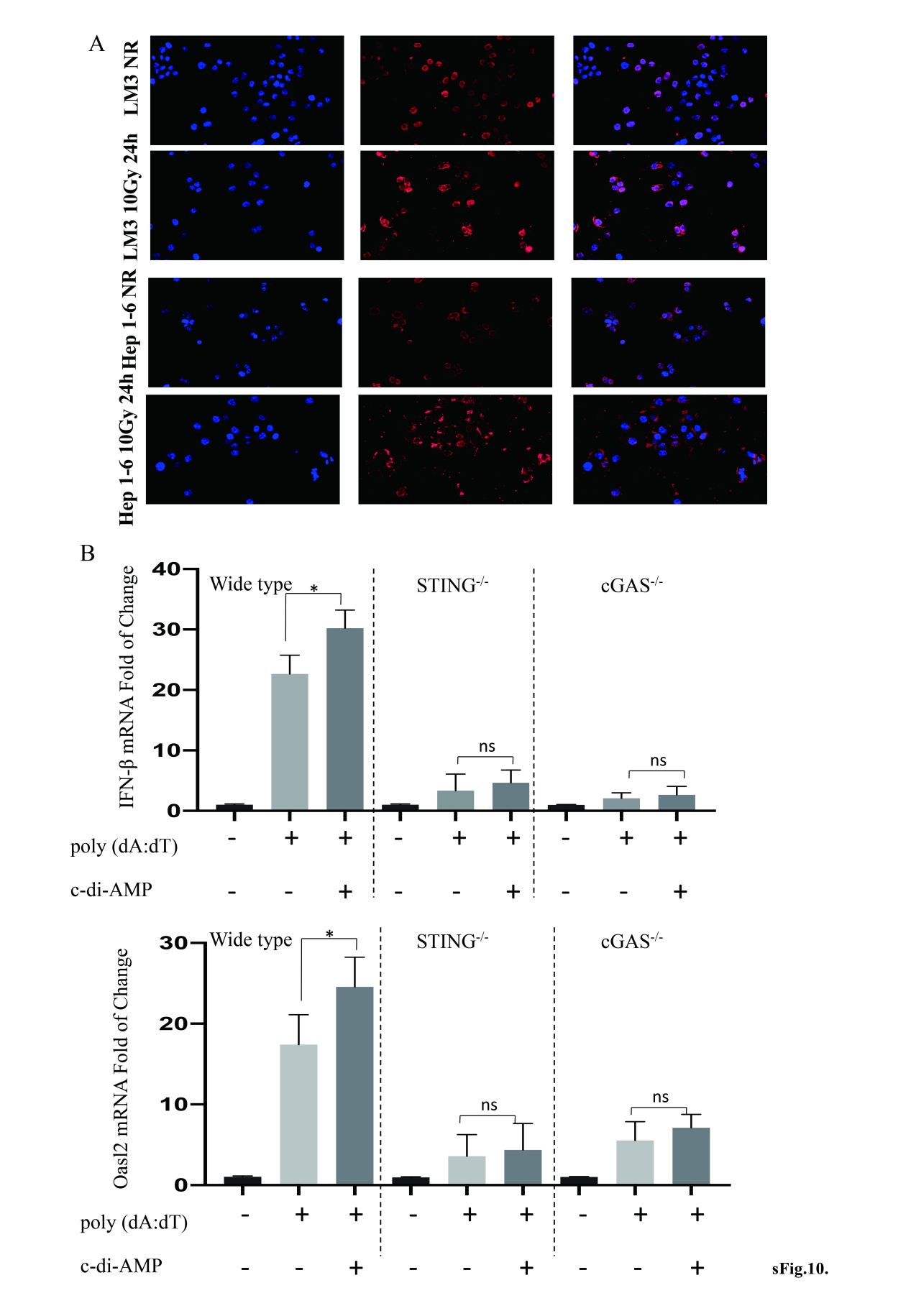


**Supplementary Figure 10: Secretion of dsDNA induced by radiation plays a synergistic role with c-di-AMP in anti-tumor immune regulation** Human HCC cell line (HCCLM3) and mouse HCC cell lines (Hepa 1-6) were exposed to 8 Gy radiation, cytosolic dsDNA (A) was detected by immunofluorescence after 24 h. (B) BMDCs from wild type, cGAS-/- and STING-/- were co-cultured with poly (dA:dT) (1 ug/ml, delivered by Lipofectamine 2000 ), c-di-AMP, or combined for 24 h. mRNA levels of IFN-β and Oasl2 were quantified by qRT-PCR.
